# Our role in physicians' education

**DOI:** 10.1590/S1679-45082013000200001

**Published:** 2013

**Authors:** Flávio Takaoka, Júlio César Monte, Cláudio Schvartsman

**Affiliations:** Hospital Israelita Albert Einstein, São Paulo, SP, Brazil

Including a medical residency program at a philanthropic hospital with a clinical team open to innovation, such as the one at *Hospital Israelita Albert Einstein* often prompts questions about the role of the physicians on our team and the academic community in physician education.

How can this role be justified amid the continuing increase in residency programs for new specialties?

In 2004 we implemented our first residency program in adult intensive care. Today, 9 years later, we have 74 residents training in 11 active residency programs. In addition, three new residency specialties will be launched in 2014: anesthesiology, family and community medicine, and clinical pathology.

Our institution's vision and values^*^, which are aligned with hospital daily practices, enable communication with our colleagues. Social practice, education, and good actions are seen in the care of underserved patients who benefit from the Paraisópolis Community Program supported by our hospital. We also offer an Organ Transplantation Program in partnership with the Ministry of Health, along with several other educational and patient care programs.

It is important to emphasize that care excellence is but one of our major pillars. Generation of knowledge from our institution is another equally important pillar, structured and supported by *Instituto Israelita de Ensino e Pesquisa* (IIEP). In 2012, with the support of IIEP, 202 original articles were published in indexed journals – a growth of 15% compared with 2011.

The presence of residents suggests the existence of updated protocols and well-defined indicators that benefit the organization's structure and medical team. These, in turn, often improve the quality and safety of care for patients. As a result, the responsible physician begins to include more updated routines as scientific knowledge moves forward. Attending physicians understand that the educational process of clinical cases discussion and other training activities for residencies helps improve care delivery for patients.

Our institution has competent and committed instructors who both teach and conduct research. Our care programs provide excellent and broad patient coverage. We trust that residents attending our programs will become successful specialists in their areas of choice. However, this alone is not enough to educate good physicians.

Our residency programs emphasize the essential values of professionalism and humanism to the new generation of physicians-in-training. Such values are threatened by other values in modern society and by new approaches, such as treating medicine as a business. On the other hand, new economic mechanisms are becoming necessary for managing a medical practice; therefore, physicians require knowledge on cost-effectiveness and pharmacoeconomics, among others. All these changes could frustrate the expectations of a new generation of physicians with regard to social relationship and personal values. Our commitment to this new generation is not to let them turn patients into customers, trust into suspicion, values into margins, or physicians into providers.

The *Hospital Israelita Albert Einstein* has wellestablished principles, vision, knowledge, staff, and structure. We believe our institution not only has the ability to receive new physicians-in-training but, moreover, *must* participate in the education of this new generation of physicians.

## Footnotes

*
http://www.einstein.br/sobre-a-sociedade/Paginas/missao-visao-e-valores.aspx

